# Low-pressure pulsed focused ultrasound with microbubbles promotes an anticancer immunological response

**DOI:** 10.1186/1479-5876-10-221

**Published:** 2012-11-11

**Authors:** Hao-Li Liu, Han-Yi Hsieh, Li-An Lu, Chiao-Wen Kang, Ming-Fang Wu, Chun-Yen Lin

**Affiliations:** 1 Department of Electrical Engineering, Chang-Gung University, Taoyuan, Taiwan; 2 Department of Hepatogastroenterology, Chang-Gung Memorial Hospital, Taoyuan, Taiwan; 3 Animal Medicine Center, National Taiwan University, Taipei, Taiwan

## Abstract

**Background:**

High-intensity focused-ultrasound (HIFU) has been successfully employed for thermal ablation of tumors in clinical settings. Continuous- or pulsed-mode HIFU may also induce a host antitumor immune response, mainly through expansion of antigen-presenting cells in response to increased cellular debris and through increased macrophage activation/infiltration. Here we demonstrated that another form of focused ultrasound delivery, using low-pressure, pulsed-mode exposure in the presence of microbubbles (MBs), may also trigger an antitumor immunological response and inhibit tumor growth.

**Methods:**

A total of 280 tumor-bearing animals were subjected to sonographically-guided FUS. Implanted tumors were exposed to low-pressure FUS (0.6 to 1.4 MPa) with MBs to increase the permeability of tumor microvasculature.

**Results:**

Tumor progression was suppressed by both 0.6 and 1.4-MPa MB-enhanced FUS exposures. We observed a transient increase in infiltration of non-T regulatory (non-Treg) tumor infiltrating lymphocytes (TILs) and continual infiltration of CD8+ cytotoxic T-lymphocytes (CTL). The ratio of CD8+/Treg increased significantly and tumor growth was inhibited.

**Conclusions:**

Our findings suggest that low-pressure FUS exposure with MBs may constitute a useful tool for triggering an anticancer immune response, for potential cancer immunotherapy.

## Background

The potential use of focused ultrasound for cancer treatment has long been recognized. Continuous-wave (CW) high-intensity focused ultrasound (HIFU) is a particularly promising approach to rapidly produce high temperatures at the target site, resulting in necrosis of tumor cells [1-4]. Ultrasonic energy emitted from concave piezoelectric ceramics can be tightly focused with radial and axial dimensions of only 1–2 mm and 10–20 mm, respectively, based on the range of frequencies and transducer geometry, so multiple sonications are required to achieve complete tumor ablation [2-6]. CW-HIFU has been successfully applied for non-malignant-cancer treatment, such as partial ablation of uterine fibroids for symptomatic relief [4,7], relief of bone-metastasis pain [8,9], or preliminary high-precision targeted ablation in the brain [3,10,11]. Medical imaging can be used to guide ablation of malignant solid tumors in breast cancer [12,13], prostate cancer [14], pancreatic cancer [6], or liver cancer [13,15], by introduction of multiple “cigar”-shaped thermal lesions to avoid leaving any residual tumor behind.

Mounting evidence suggests that in addition to thermal ablation of cancer cells, HIFU may also boost the host antitumor immune response including CD4+/CD8+ related tumor-infiltrating lymphocytes (TILs) [16,17], dendritic cells (DCs) [18], and antigen presenting cells (APCs)[19]. This effect could potentially reduce local tumor recurrence and metastasis, especially in patients with low antitumor-immunity or inaccessible tumors. In addition to direct ablation by CW-HIFU, some studies have demonstrate that pulsed-mode HIFU with negative pressures equal or higher than that used for thermal ablation (7–12 MPa) boosts the systemic antitumor immune response through activation and maturation of DCs or APCs [20,21]. Although pulsed-HIFU and CW-HIFU rely on different cell-killing mechanisms, both induce local tumor debris which contains tumor antigens or heat-shock proteins that could directly or indirectly enhance the infiltration of DCs [22].

Recent reports suggest that the permeability of blood vessels can be greatly increased by focused ultrasound in the presence of microbubbles (MB). These MBs can be used to enhance targeted delivery of chemotherapeutic agents, even at greatly reduced acoustic pressures only slightly above the pressure threshold employed in diagnostic ultrasound [23-25]. The enhanced permeability could conceivably also result in boosting the antitumor immune response by altering the tumor microenvironment, enhancing chemokine/cytokine release from tumor cells, or increasing infiltration of immune cells such as tumor-infiltrating lymphocytes (TILs) [26]. Currently little is known about a potential antitumor immune response upon treatment with low-pressure FUS and MBs.

Here we investigated the immunological changes induced by MB-enhanced, low-pressure FUS using a CT-26 tumor murine model. We found that pulse-mode low-pressure FUS exposure in the presence of MBs (denoted as MB-FUS) could trigger a significant anticancer immune response and significantly suppressed tumor progression.

## Methods

### Mouse subcutaneous tumor model

BALB/c mice purchased from the the National Laboratory Animal Center were bred in the animal house of Chang Gung University and were used in experiments at age 8–10 weeks. A total of 280 animals were used in this study. All animal experiments were approved by the Animal Committee and conformed to the experimental animal care guidelines (IACUC Approval No. CGU11-058; Chang-Gung University, Taoyuan, Taiwan). CT26 cells were cultured at 37°C in a humidified 5% CO_2_ atmosphere in RPMI1640 medium (GIBCO, CA, USA) containing 0.25% D-glucose, 0.15% sodium bicarbonate, 10 mM HEPES, 10 mM sodium pyruvate, 100x diluted antibiotic-antimycotic and 10% complement-inactivated (56°C for 30 minutes) fetal bovine serum (FBS). A tumor model was prepared by injecting 2×10^5^ CT-26 tumor cells suspended in 100 μl phosphate buffered saline (PBS) subcutaneously into the right hindlimb of BALB/cByJNarl mice. The tumor was allowed to grow for 9–11 days to reach a maximum diameter of 8–11 mm before FUS treatment.

### Setup of Sonogram-guided MB-FUS exposure

**Figure 1:**
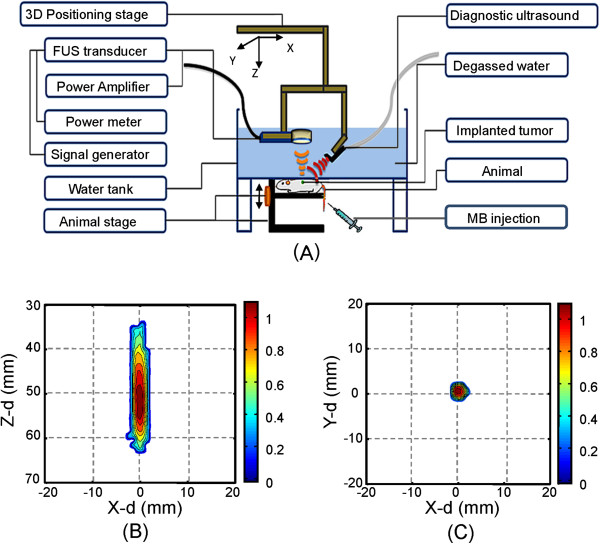
**Experimental setup and measured FUS field.** (**A**) Experimental setup of sonogram-guided FUS exposure; )**B, C**) measured focused ultrasound pressure distribution along the radial direction and beam axis.

The experimental setup of the sonogram-guided MB-FUS exposure system is illustrated in Figure 1. A focused ultrasound transducer (Sonic Concepts, Seattle, WA, USA; operating frequency = 0.5 MHz, active element diameter = 64 mm, radius curvature = 55 mm) driven by an arbitrary function generator (33220A, Agilent, Palo Alto, CA, USA) with a radio-frequency power amplifier (150A100B, Amplifier Research, Souderton, PA, USA) for RF signal amplification and a power meter (Model-4421, Bird, USA) for electrical power sensing was used. The focused ultrasound transducer was mounted on top of a plexiglass chamber filled with degassed water to generate a focal beam passage. FUS exposure was controlled by an in-house graphic user interface based on the Labview program and power feedback was monitored through a GPIB interface on a laptop computer. An in-house manufactured three-axis stepping-motor positioning system was integrated and controlled by the same graphic interface. Moreover, to ensure the alignment of the focal exposure with the tumor site, a laptop-computer based ultrasound imager (T- 3000, Terason Inc., Burlington, MA, USA; imaging frequency = 5 MHz) was integrated in the same interface to provide real time co-registered B-mode tumor section imaging along with the pre-calibrated FUS target points. The imaging plane was tuned to co-localize with the focal beam position during the co-registration step, then the positions of imaging and FUS probe were fixed so that the FUS focus appeared at a fixed point on the imaging frame during the entire treatment procedure. The measured pressure field of the ultrasound transducer is shown in Figures 1B and 1C, and the measured -3dB dimensions were about 18 and 3 mm, respectively.

Animals were anesthetized with isofluorane (2.5-3% volume with oxygen). The animal was placed on the acrylic holder and the skin flanking the tumor was fastened and fixed by stainless clips. The top of the tumor was attached to the bottom of the filled water tank which had a 50×50-mm^2^ opening. This opening was sealed with an acoustic-energy permeable membrane and a 3-mm-thick gelatin pad to insure perfect coupling with no intervening gas. FUS exposure was delivered at 5 and 30 W of electrical power, equivalent to measured acoustic negative-peak pressures of 0.6 and 1.4 MPa, respectively. Before focused ultrasound exposure, a 0.1 mL/kg bolus of SonoVue MBs (Sonovue, Bracco Diagnostics Inc., Milan, Italy) mixed with 0.2 mL of saline was injected intravenously (IV), followed by flushing with 0.2 mL heparin. Immediately after MB bolus injection (typically within 10 seconds), burst-mode FUS energy was delivered to the animal (burst length = 100 ms, pulse repetitive frequency = 1 Hz, sonication duration = 20s; parameters were selected based on our previous experience in increasing brain tissue vascular permeability [27]). Multiple sonications were carried out to completely cover the tumor. Animals typically underwent 9 to 12 sonications to allow coverage of the entire tumor region (range of total exposure time 180s to 240s). Since the focal dimension was measured as approximately 3 mm along the radial direction, the spacing between individual adjacent focal positions was set at 3 mm. A total of 280 animals were used in this study (Additional file 1: Table S1). Twelve animals were used to test tumor vascular permeability by injecting FITC-dextrans; 99 animals were assigned to observation of tumor progression; and 45, 30 and 71 animals were sacrificed 1, 3, and 18 days after MB-FUS exposure, respectively, to evaluate TIL infiltration. An additional 23 animals were used to confirm histological changes.

### Temperature monitoring

To rule out temperature elevation as a confounding factor, temperature measurements were conducted in a tissue-mimicking phantom using the same FUS exposure energy. A single-point thermocouple (T-type, diameter=0.05 mm, thin-epoxy coating and bare tip) was inserted into the phantom so that the tip colocalized with the focal spot. The thermocouple was connected to a thermometer system (TC-2190, National Instruments, Austin, TX, USA) for temperature acquisition. Temperature measurements were performed with a sampling frequency of 1 Hz using a PC connected to a GPIB interface.

### Isolation of TILs

Tumors from day 1, 3 and 18 tumor-bearing mice were chopped into small pieces using a razor blade and 1g of tumor was incubated with 10 ml collagenase type IV (1mg/ml; GIBCO, CA, USA) in PBS buffer on a shaking incubator (100 rpm) at 37°C for 30 min. Cells were passed through nylon mesh, centrifuged at 1800 rpm for 3 minutes, and washed with RPMI1640 medium. Cell pellets were re-suspended in 8 ml RPMI1640 medium and layered over 4 ml Ficoll (Pharmacia, Peapack, NJ) in a 15-ml centrifuge tube. After centrifugation at 2000 rpm for 20 minutes with the deceleration speed set at 2, the single-cell suspension was separated from the Ficoll, and the leukocytes were recovered from the interface.

### Antibodies and flow cytometry

Anti-CD4-PE, anti-CD45-PE, anti-Foxp3-APC, anti-CD8-APC, anti-FceRIa-APC, anti-CD117-FITC and anti-CD45-FITC antibodies were used for intracellular staining. TILs were washed twice with Hank’s balanced salt solution (HBSS), then fixed and permeabilized in Fix/Perm buffer according to the manufacturer’s instructions for 30 min. Cells were washed twice with permeabilization buffer and then incubated with appropriate antibodies at 4°C for 30 min in the dark. Unbound antibodies were removed by washing twice with permeabilization buffer. Flow cytometry analyses were performed on a three-color fluorescence FACScalibur cytometer using CellQuest software (Becton-Dickinson, CA, USA).

### Histological examination

To confirm FUS-induced blood vessel permeability enhancement, FITC-labeled dextran (60 kDa; 500 μL, 0.5 mg/mL; Santa Cruz Biotechnology, USA) was injected IV. Animals were sacrificed 2 hours after FUS treatment. Paraformaldehyde-fixed and paraffin-embedded tumors were used to prepare 10 μm-thick sections for fluorescence observation. Adjacent sections were stained with hematoxylin-eosin (HE) to observe histological changes after MB-FUS exposure. Terminal deoxynucleotidyl transferase (TdT)-mediated dUTP-biotin nick end labeling (TUNEL) was conducted to detect apoptotic DNA damage using the Apo-Brdu-IHC in situ DNA fragmentation assay kit (BioVision, Mountain View, CA). Immunohistochemical (IHC) staining with anti-heat-shock-protein 60 (hsp60) antibody (dilution 1:200, Cat. No. SC-376261, Santa Cruz Biotechlogy, Santa Cruz, CA, USA) was also performed to investigate cell-damage- or stress-induced heat-shock-protein regulation. Animals were sacrificed 3 days after MB-FUS exposure. Ten micrometer sections from paraformaldehyde-fixed, praffin-embedded tumors were prepared for TUNEL and hsp60 IHC staining.

### Statistical analysis and tumor volume measurement

Flow-cytometric data were displayed as means ± standard deviations. The Mann–Whitney U test was used for statistical analysis of differences between groups. Calculations were performed with PRISM (version 5.00; GraphPad Software). Differences were recognized as statistically significant at *p* < 0.05.

Tumor volume was assessed primarily by vernier caliper. Tumor volume was calculated as LxW^2^x0.52, where L was the longest diameter and W was the diameter perpendicular to L. Tumor volume was measured every third day after treatment, ending at 25 days post tumor implantation. To verify the accuracy of caliper-measured volume, some animals were subjected to magnetic resonance imaging (MRI) to acquire high-resolution images of tumor anatomy (field strength = 3 Tesla, Trio with Tim, Siemens, Erlangen, Germany). Tumor volume was quantified by analyzing T2-weighted images with the following parameters: TR/ TE = 3490 ms/ 98 ms, matrix size= 144 × 256, FOV = 47 × 83 mm, slice thickness = 1 mm. The MRI-based tumor measurements were found to be comparable to the caliper-based estimates (data not shown).

## Results

### Sonogram-guided Microbubble-enhanced FUS exposure induces local enhancement of microvascular permeability within a tumor

**Figure 2:**
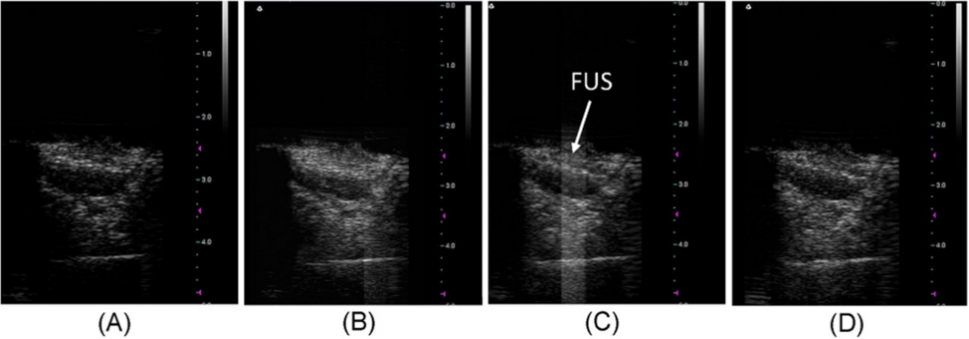
**Temperature measurements of FUS exposure.** Measured temperature elevation for 1.4-MPa FUS with 100-ms burst length in a tissue-mimicking graphite phantom (with and without MBs). Continuous mode (CW) sonication at 1.4-MPa was performed for comparison (also with and without MBs).

Sonographically-guided MB-FUS exposure can be focused by careful calibration to deliver focal energy to a specific area of the tumor mass. We observed an increase in the image contrast of the entire tumor mass after injection of MBs into the animal (Figure 2A and 2B), demonstrating that MBs could circulate within the tumor. After multiple MB-FUS exposures to cover the whole tumor (Figure 2C and 2D; sonication duration 180s, 3×3 target positions), the signal intensity did not show apparent decay, implying that most of the MBs remained intact and in the vicinity of the tumor.

**Figure 3:**
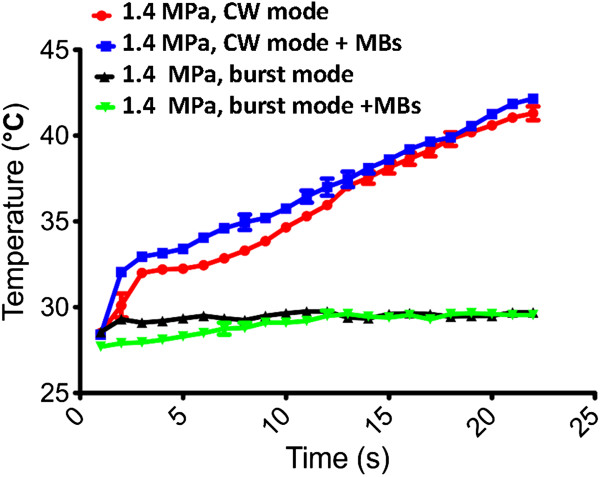
**Typical ultrasound images for real-time tumor position monitoring.** (**A**) Before injection of microbubbles; (**B**) after microbubble injection but prior to FUS sonication; (**C**) after microbubble injection and during FUS exposure; (**D**) after completing FUS exposure. Arrow in (**C**) marks the FUS energy exposure that produced interference in the ultrasound image.

Figure 3 shows the measured temperature elevation resulting from 1.4-MPa FUS exposure (continuous- or pulsed-mode) alone or in the presence of MBs. Although temperature buildup was observed for continuous-mode 1.4-MPa exposure (both with or without MBs), the burst mode exposure in the presence of MBs used in this study did not cause any increase in temperature and we could therefore exclude a thermally-induced biological effect.

**Figure 4:**
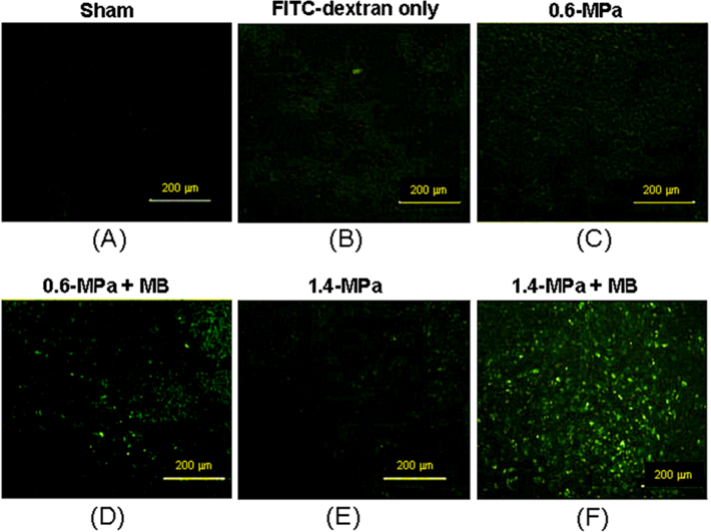
**Fluorescence microscopy of FUS exposure locations.** Microscopic images of fluorescence produced by FITC-dextran in sections of dissected tumors from animals that were sacrificed on the 10^th^ day after tumor implantation. (**A**) Animal without FITC-dextran injection (sham). All other panels show data from animals that were injected with FITC-dextran. (**B**) Unsonicated animal (control); (**C**) 0.6-MPa FUS exposure alone; (**D**) 0.6-MPa FUS exposure with microbubbles; (**E**) 1.4-MPa FUS exposure alone; (**F**) 1.4-MPa FUS exposure with+ microbubbles.

Fluorescence microscopy revealed that injected 60-kDa FITC-labeled dextran (Figure 4B) did not leak from the tumor vasculature by comparison to an animal that was not injected with fluorescent dextran (Figure 4A). FUS exposures of 0.6 or 1.4 MPa in the absence of microbubbles similarly did not lead to enhanced permeability of fluorescent dextran (Figure 4C and 4E). However, in the presence of microbubbles, we observed dramatic leakage of FITC-labeled dextran into the extravascular tumor spaces, with significantly more leakage for 1.4-MPa than 0.6-MPa FUS (Figure 4F vs 4D). Detailed results of fluorescence signal enhancement are presented in Additional file 1: Table S2.

**Figure 5:**
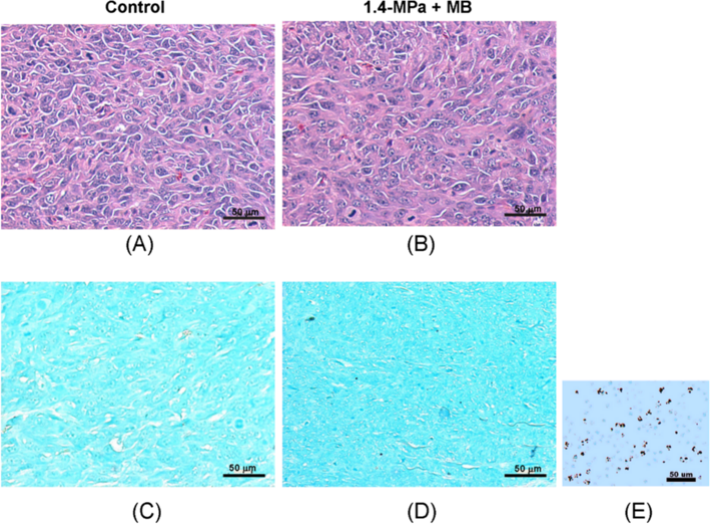
**Histology of FUS exposure locations.** (**A, B**) HE stains (40×) in control and microbubble-enhanced 1.4-MPa FUS exposure. Bar = 50 μm. (**C, D**) TUNEL stain to detect apoptosis (40×) for control and microbubble-enhanced 1.4-MPa FUS exposure, and (**E**) is a positive TUNEL stain as a reference to (C) and (D). Bar = 50 μm.

Histological examinations were performed to determine whether FUS exposure was associated with cell necrosis or apoptosis. First, HE staining revealed no obvious tissue damage for 1.4-MPa FUS exposure without MBs (Figure 5A). In contrast, HE staining for 1.4-MPa FUS in the presence of MBs revealed enlarged vascular/cellular or extracellular spaces with some local erythrocyte extravasations, indicating sufficient blood vessel permeability to allow leakage of micrometer-sized molecules, erythrocytes or other cells (Figure 5B). Second, there was no apparent increase in apoptotic cells in the tumor region after 1.4-MPa MB-presented pulsed-mode FUS exposure (Figure 5D) compared to the control (Figure 5C), indicating that this FUS exposure did not trigger acute cell apoptosis. Overall, the histological evidence supported enhanced tumor-tissue permeability without a marked increase in cellular damage by MB-enhanced pulsed FUS exposure at pressures up to 1.4 MPa.

### Microbubble-enhanced FUS exposure is associated with an increase in host antitumor immune response

**Figure 6:**
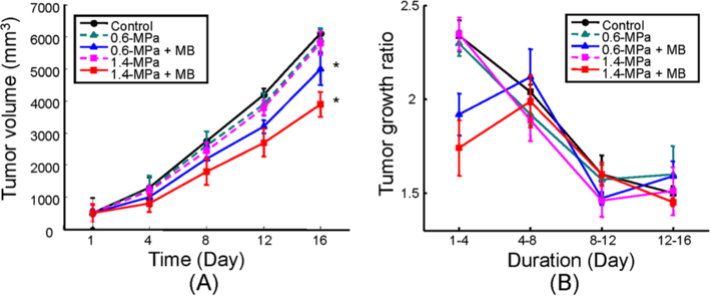
**Tumor progression analysis.** (**A**) Tumor progression from day 1 to 16 (after FUS exposure) for different FUS exposure conditions; (**B**) Tumor progression ratios for four different time periods (time period 1–4, 4–8, 8–12, and 12–16 days after FUS exposure, respectively). FUS exposures were conducted on day 10.

Next we investigated the influence of MB-FUS exposure on tumor growth. In the absence of MBs, we did not observe any significant changes in tumor progression with either 0.6 or 1.4-MPa FUS exposure compared to the control (no FUS) group (Figure 6A). However, MB-enhanced FUS sonication resulted in an 18.1% and 34.4% reduction in tumor volume for 0.6-MPa and 1.4-MPa FUS, respectively, compared to control (p < 0.05 in both cases).

The tumor growth ratio was defined as the ratio of the tumor volumes of the 2^nd^ and 1^st^ time points, and was calculated for each two consecutive time points (Figure 6B). We noted that the most significant inhibition of tumor growth progression occurred within the first three days; The tumor growth ratio decreased from 2.3 to 1.9 and 1.74, respectively, for 0.6 and 1.4-MPa MB-FUS exposure (Figure 6B). No reduction in the tumor growth ratio was observed for 0.6 or 1.4-MPa FUS without MBs. These data suggested that MB-FUS-exposure resulted in an antitumor effect mainly within the initial 72 hours after treatment.

### Microbubble-enhanced FUS exposure provides local modulation of the immune environment

**Figure 7:**
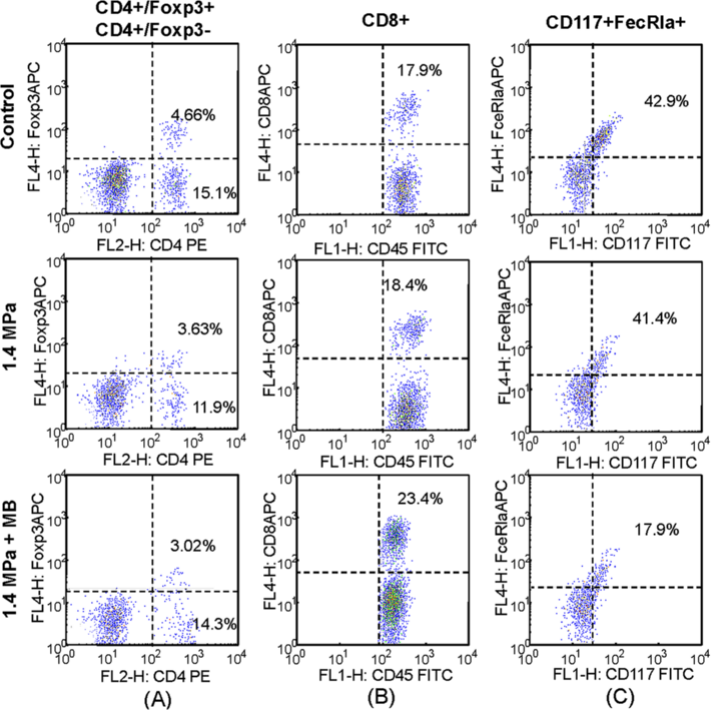
**Typical flow cytometry (day 18 after FUS exposure).** Relative FITC-labeled dextran leakage into the tumor regions after FUS exposure. Total area = FITC-fluorescence leakage area in the section; Area fraction = fraction of the FICT-fluorescence area of the whole section. Naive = tumor observation without FUS or FITC-dextran injection. Control = tumor observation with FITC-dextran injection only.

**Figure 8:**
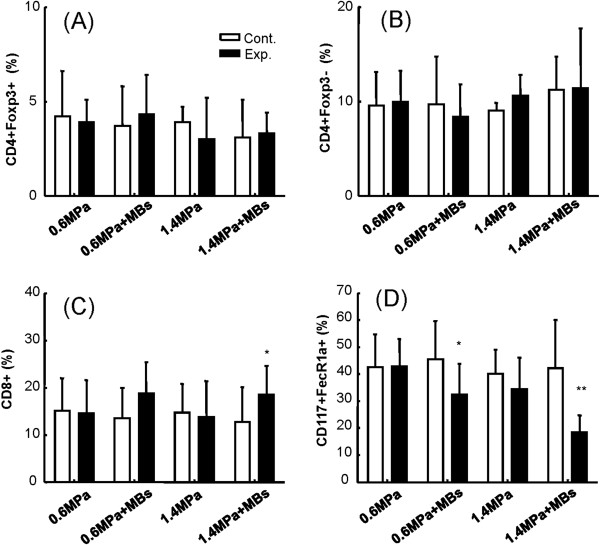
**Flow cytometric analysis of FUS exposure region (day 18 after FUS exposure).** Flow cytometric analysis of the change in TIL populations under different FUS-exposure conditions (0.6-MPa, 0.6-MPa with MBs, 1.4-MPa, and 1.4-MPa with MBs, respectively) on day 18 (Exp.; black bars) compared to untreated controls (Cont.; white bars). (**A**) Foxp3^+^CD4^+^/CD45^+^ TILs; (**B**) Foxp3^+^CD4^-^/CD45^+^ TILs; (**C**) CD8^+^/CD45^+^ TILs; (**D**) CD117^+^FceRIa^+^/CD45^+^ TILs. (* indicates p < 0.05; ** indicates p < 0.005).

The significant reduction in tumor progression by 1.4-MPa MB-FUS prompted us to investigate possible immunological changes in the tumor microenvironment at different time points. First, we investigated a possible correlation between tumor progression (Figure 6) and TIL infiltration into the tumor regions. Typical flow cytometric analyses using different FUS exposure parameters are shown in Figure 7, and cell percentages are shown in Figure 8. The infiltrating CD4+CD8+ cells, representing cytotoxic T lymphocyte (CTL) subgroups, were significantly increased with 1.4-MPa MB-FUS (p < 0.05). Exposure to 0.6-MPa MB-FUS also showed an average increase in CTLs but without statistical significance. On the other hand, CD117+FecR1a+ TILs, representing the Mast cells subgroups, underwent a remarkable population drop for both 0.6- and 1.4-MPa MB-FUS exposure.

**Figure 9:**
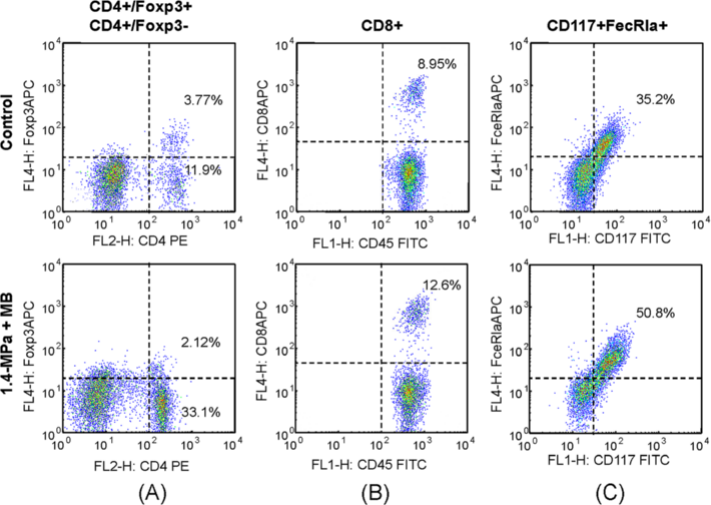
**Typical flow cytometry after FUS exposure.** Typical flow cytometric analysis to demonstrate cell populations in untreated (upper panel) and microbubble-enhanced 1.4-MPa FUS treated (lower panel) groups at day 1 after FUS exposures. (**A**) CD4^+^Foxp3^+^ (i.e., Treg cells) and CD4^+^Foxp3^-^ TILs; (**B**) CD8^+^/CD45^+^ TILs (i.e., cytotoxic T lymphocytes); (**C**) CD117^+^FceRIa^+^/CD45^+^ TILs (mast cells).

**Figure 10:**
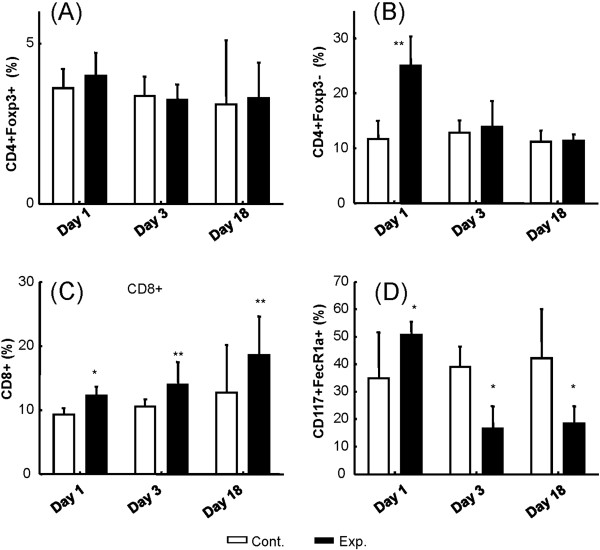
**Flow cytometric analysis of FUS exposure region.** Flow-cytometric analysis of TIL subtype populations at different time points (1, 3, and 18 days after FUS exposure), for the untreated control group (Cont., white bars; defined as animals without MB-presented FUS exposure) compared to the MB-enhanced 1.4-MPa FUS treated group (Exp., black bars). (**A**) Foxp3^+^CD4^+^/CD45^+^ TILs; (**B**) Foxp3^+^CD4^-^/CD45^+^ TILs; (**C**) CD8^+^/CD45^+^ TILs; (**D**) CD117^+^FceRIa^+^/CD45^+^ TILs. (* indicates p < 0.05; ** indicates p < 0.005).

Next, since control of tumor progression was most profound in the acute stage, we investigated TIL infiltration at one or three days after FUS exposure in the control and 1.4-MPa MB-FUS groups. A typical flow cytometric analysis showing changes in the cell populations of untreated and 1.4-MPa MB-FUS treated groups one day after FUS exposure is presented in Figure 9. The percentages of different cells within the untreated control group and the 1.4-MPa MB-FUS treated group were compared on days 1, 3 and 18 (Figure 10). The percentage of Treg cells (CD4+Foxp3+T cells) in the tumor microenvironment was similar at different time points (Figure 10A). In contrast, the percentage of non-Treg cells (CD4+Foxp3-T cells) underwent a temporal surge within 24 hours after MB-FUS exposure, and then decreased to a level similar to the control group on day 3 and 18 (Figure 10B). Furthermore, the percentage of CD8+ cells gradually increased over time in the treated group compared to the control (Figure 10C). Interestingly, the percentage of mast cells showed an abrupt initial increase on day one but a decrease on days 3 and 18 (Figure 10D).

**Figure 11:**
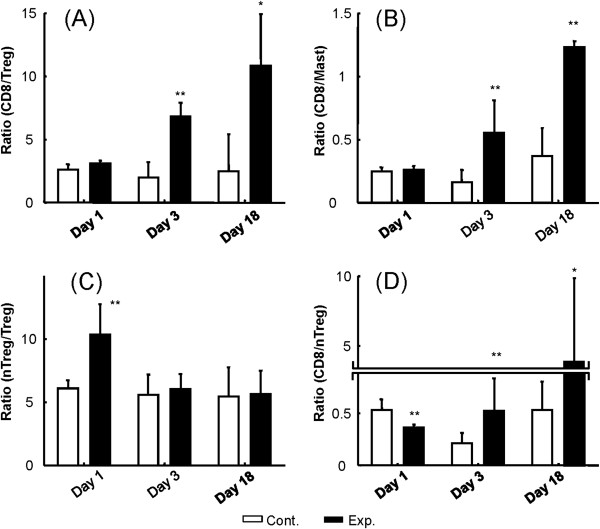
**Flow cytometric analysis of FUS exposure region.** Flow-cytometric analysis of the ratios of two TIL subtypes at different time points (1, 3, and 18 days after FUS exposure), for the untreated control group (Cont., white bars; defined as animals without MB-presented FUS exposure) compared to the MB-enhanced 1.4-MPa FUS treated group (Exp., black bars). (**A**) Ratio of CD8 and CD4^+^Foxp3^+^ TILs; (**B**) ratio of CD8 and CD117^+^FceRIa^+^/CD45^+^ TILs (mast cells); (**C**) ratio of CD4^+^Foxp3- and CD4^+^Foxp3^+^ TILs; (**D**) ratio of CD8 and CD4^+^Foxp3- TILs. FUS exposure was set to 1.4-MPa of pressure, in the presence of microbubbles. (* indicates p < 0.05; ** indicates p < 0.005).

Treg and possibly mast cells play immune inhibitory roles, and CD8+T cells may act as effectors in the tumor microenvironment. We therefore examined the changes in the ratios of CD8+T cells/Treg cells and CD8+T cells/mast cells. These ratios were similar to the control (untreated) group on day 1 but increased significantly on day 3 and day 18 (Figure 11A and 11B). Conversely, non-Treg cells could possibly contribute to the function of CD8+T cells. We thus examined the ratio of non-Treg to Treg cells in the tumor microenvironment, and found that this ratio increased on day 1 but then returned to a level similar to the control group (Figure 11C). The ratio of CD8+Tcells/non-Treg cells decreased on day 1 and then increased significantly on day 3 and day 18 (Figure 11D). In summary, we found that treatment with 1.4-MPa MB-FUS was associated with dramatic changes in the percentages of different immune cells in the tumor microenvironment and correlated with significantly inhibition of tumor growth, apparently mediated by this immune response, especially during the first three days after treatment.

## Discussion

**Figure 12:**
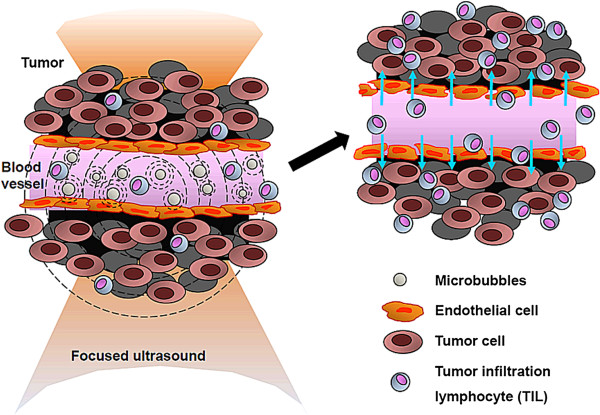
**Model of immunological response triggered by microbubble-facilitated focused ultrasound exposure.** Exposure of tumor tissue to FUS in the presence of MBs significantly enhances blood-vessel permeability, changing the microenvironment of the tumor tissue region, and enhancing the recruitment and penetration of tumor-infiltrating lymphocytes into the tumor tissue.

It is well known that FUS exposure in the presence of microbubbles can increase vascular permeability in various organs or tumor tissues [23,28-31]. A previous study by Miller *et al.*, in the absence of immunological observations, demonstrated a profound tumor suppression effect within 4 days caused by MB-presented FUS exposure [32]. They hypothesized that, rather than thermal effects, enhanced cavitation was primarily responsible for tumor suppression. In this study, we confirmed that pulsed-mode MB-presented FUS exposure significantly enhanced leakage of 60-kDa FITC-dextrans into tumor tissues, thereby providing direct evidence of an increase in tumor vascular permeability (Figure 4). In addition, we observed significant and concurrent infiltration of immune cells, particularly TILs, into the tumor regions (Figures 9,11). Since enhancement of permeability is the main recognized biological effect of MB-presented pulse-mode FUS exposure, we hypothesized that the increase in tumor permeability caused by MB-enhanced cavitation may directly modulate the tumor microenvironment, leading to an increase in tumor cytokine/chemokine release and triggering TIL infiltration (Figure 12). However, we note that the relationship between enhancement in permeability caused by MB-FUS exposure and the observed immunological response remains unknown and needs to be further investigated.

One possible underlying mechanism to explain the changes in tumor permeability or microenvironment and TIL infiltration in response to MB-FUS is that MB-FUS exposure may trigger the production of heat-shock protein (hsp) or other immunomodulatory factors (so-called danger signals) which could in turn trigger TIL infiltration. Previous studies demonstrated that hsp is not only induced by heat, but can also be triggered by mechanical stress [33,34]. Kruse *et al.* demonstrated that hsp70 overexpression is sometimes observed within a similar pressure range (intensity = 53 W/cm^2^) and a short duration (1s) of ultrasound exposure without temperature elevation [35], similar to induction caused by heat from HIFU exposure [22]. We therefore evaluated hsp60 expression and found that hsp60 indeed increased in regions of FUS exposure compared to control tumor regions, suggesting that induction of hsp overexpression may be involved in triggering of the immune response by MB-FUS (Additional file 1: Figure S1).

Besides expression of hsp or release of other danger signals, vascular disruption caused by FUS exposure may also be directly or indirectly involved in the observed tumor suppression. Previous reports showed that direct damage of the tumor endothelium will initiate vascular collapse, shut down tumor blood flow, and induce ischemic neoplastic cell death [36,37]. Strategies aimed at causing such vascular disruption include targeted gene therapy, antibodies to neovascular antigens, fusion proteins directed against specific endothelial cell receptors, cytokine induction, or agents that selectively target the cytoskeleton of proliferating endothelial cells [38,39]. Previous evidence demonstrated that CW-HIFU exposure can disrupt tumor blood vessels in liver cancer patients due to acoustic cavitation [40]. It is therefore possible that microvascular disruption could explain or contribute to the anticancer effect caused by our MB-facilitated FUS exposure.

Conventional CD4+T cells (CD4+Foxp3-T or non-Treg cells) are intriguing since they appear to play an important role in the development of mature CTLs [41] and long-lived functional memory CD8+T cells [42]. In a murine chronic viral infection model, CD4+T cell transfer was shown to effectively reverse the effects of exhausted CD8+T cells [43,44]. In our study MB-FUS exposure facilitated a response of mostly conventional CD4+T cells with a moderate response by CD8+T cells, and no response by Treg cells, consistent with an antitumor function of conventional CD4+T cells as borne out by tumor growth inhibition. The gradual increase in CD8+T cells at days 3 and 18 might be explained by assistance from the initial surge of conventional CD4+T cells.

Sato *et al.* reported that among ovarian cancer patients, those with a high CD8+/CD4+ ratio had significantly higher survival, whereas Tregs were associated with an unfavorable prognosis [45]. They concluded that CD8+ TILs and high CD8+/Treg ratios are associated with a favorable prognosis. However, others have reported that changes in the CD8+/CD4+ ratio were associated with a trend towards worse patient prognosis [46]. These differences may be ascribed to CD4+ cells containing both Treg and non-Treg cells that have opposite effects on CD8+ cells. In our study, inhibition of tumor growth by MB-FUS was accompanied by a significant increase in the percentage of non-Treg CD4+ and CD8+T cells and an increase in the ratio of CD8+/Treg, suggesting that changes in these cell populations within the tumor microenvironment indeed affected tumor growth.

The role of mast cells in tumor development is more complicated since they can both help and inhibit tumor development [47]. Mast cells can serve as promoters of the inflammatory response [48] but can also increase the invasion of tumor cells by degrading the extracellular matrix and promoting angiogenesis [49]. Mast cells are capable of expanding [50] and recruiting Treg cells [51], but have also been shown to abolish the suppressive functions of Treg cells [52]. The complicated roles of mast cells and the dual pattern of changes in mast cells in our study need to be further investigated to decipher their impact in the altered tumor microenvironment.

The immune-triggering effects of high-pressure CW-HIFU have been studied for nearly two decades [53]. Indeed, the immune response triggered by CW-HIFU could potentially be just as effective for cancer treatment as the thermal-ablation of cancer cells [17,19,22,54]. CW-HIFU thermal ablation of osteosarcoma, hepatocellular carcinoma, and renal cell carcinoma was found to be accompanied by a marked increase in CD4+ cells at the ablated tumor-tissue margins, but no significant changes in CD8+ or CD3+ cell populations [17]. The thermal tumor-tissue destruction caused by CW-HIFU produces tumor debris antigens [22] that attract infiltration of numerous leukocytes at the margins of the coagulated tissues [19,54], and the immune response is thus primarily mediated by cell debris and the accompanying inflammatory reaction.

More recently, mechanical pulsed-HIFU tumor-cell destruction has been shown to elicit an antitumor immune response that is similar to that of thermal-ablation CW-HIFU [19]. Hu *et al.* demonstrated that in this case increased release of endogenous ATP and hsp60 triggers secretion of interleukin-12 by DCs and TNF-α by macrophages [19]. CTLs and IFN-γ-secreting cells are also elevated after treatment [20]. In contrast to CW-HIFU or pulsed-HIFU that triggers an immune response due to cell death following thermal ablation or mechanical destruction, we showed that MB-enhanced 1.4-MPa focused ultrasound may boost the antitumor immunological response by increasing the permeability of the tumor microenvironment, in the absence of extensive cell damage.

Our data revealed the potential key role played by MBs in triggering an immune response with low-pressure FUS. Weak-pressure ultrasound exposure by itself is known to have a very limited capacity to activate an immunological response [55]. Even long exposure times of animals to CW low-pressure ultrasound (0.66 W/cm^2^; 7–60 minutes) did not affect cell viability and was associated with only a slight increase in apoptotic keratinocytes and dendritic cells without any changes in important cytokines such as IL-6 and TNF-α when the intensity was increased to 5 W/cm^2^. The mechanical index (MI) is a better indicator of cell destruction. An ultrasound intensity level of 0.66-5 W/cm^2^ translates to a MI of 0.68-1.9 [55]. We used a similar MI in our study (MI = 0.8 and 1.98 for 0.6- and 1.4-MPa 0.5 MHz ultrasound FUS exposure), although we employed the burst-tone mode with individual site exposure, which is equivalent to a total exposure time of 2s (100 ms/s × 20s). Thus it is unlikely that our FUS exposure could have triggered an immune cell response through cellular damage of tumor cells in the absence of microbubbles.

In conclusion, compared to the thermal or mechanical destructive impact of HIFU exposure that results in APC-presenting and macrophage-recruiting immunological changes, permeability-enhancing MB-FUS exposure presents a new opportunity to facilitate alterations in a tolerogenic tumor microenvironment, possibly serving as an independent therapeutic route or an effective adjuvant for current first-line cancer treatment.

Despite our promising results, we note that tumor growth was inhibited mainly during the first three days after treatment, and that more work will need to be carried out in an animal model to optimize the immune response and maximize growth inhibition. In addition, the detailed mechanism of cancer growth inhibition as mediated by changes in the tumor microenvironment and immune cell populations needs to be elucidated by further studies. However, 1.4-MPa MB-enhanced FUS is already known to be safe in human subjects, which could potentially speed the development and introduction of this potential anticancer treatment modality.

## Conclusions

In this study, we successfully demonstrated that low-pressure pulsed-mode MB-FUS exposure enhanced microvascular permeability within the tumor and boosted the antitumor immune response. We showed that alteration of the immune response after FUS exposure included the gradual increase of CD8+ CTL infiltration, a sudden surge of CD4+ non-Treg cell infiltration, and an initial increase followed by decrease of the percentage of mast cells. CD4+ Treg cell levels were unchanged. In addition, low-pressure, MB-enhanced FUS exposure significantly inhibited tumor growth, especially in the initial days following treatment.

## Competing interests

The authors have no competing interests to declare.

## Authors’ contributions

HLL and ZH conducted the study and participated in data interpretation. HYH and LAL performed the statistical analysis, and wrote the manuscript. HYH and LAL participated in the in vitro studies and data analysis. HLL, HYH, LAL, CWK, CYL, and MFW participated in study design, coordination, data interpretation and writing of the manuscript. All authors read and approved the final manuscript.

## Supplementary Material

Additional file 1 **Figure S1.** Heat-shock protein 60 (hsp60) overexpression observed in IHC stain (10X) is induced by microbubble-presented focused ultrasound exposure. **Table S1.** Summary of animal experiments. In the experimental groups with 0.6-MPa or 1.4-MPa FUS exposure in the absence of MBs, animals were only evaluated on day 18 after FUS exposure. TIL = tumor infiltrating lymphocyte. **Table S2.** Relative FITC-labeled dextran leakage into the tumor regions after FUS exposure. Total area = FITC-fluorescence leakage area in the section; Area fraction = fraction of the FITC-fluorescence area of the whole section. Naive = tumor observation without FUS or FITC-dextran injection. Control = tumor observation with FITC-dextran injection only. Click here for file
