# Supplementary material for: Low-pressure pulsed focused ultrasound with microbubbles promotes an anticancer immunological response
Source: J Transl Med. 2012 Nov 11;10:221. doi: 10.1186/1479-5876-10-221 (PMC3543346; doi:10.1186/1479-5876-10-221)
Supplement: Additional file 1 — Figure S1. Heat-shock protein 60 (hsp60) overexpression observed in IHC stain (10X) is induced by microbubble-presented focused ultrasound exposure. Table S1. Summary of animal experiments. In the experimental groups with 0.6-MPa or 1.4-MPa FUS exposure in the absence of MBs, animals were only evaluated on day 18 after FUS exposure. TIL = tumor infiltrating lymphocyte. Table S2. Relative FITC-labeled dextran leakage into the tumor regions after FUS exposure. Total area = FITC-fluorescence leakage area in the section; Area fraction = fraction of the FITC-fluorescence area of the whole section. Naive = tumor observation without FUS or FITC-dextran injection. Control = tumor observation with FITC-dextran injection only. [file 1479-5876-10-221-S1.doc]

**SUPPLEMENTARY MATERIALS**

**Title:** Low-Pressure Pulsed Focused Ultrasound with Microbubbles Promotes an Anticancer Immunological Response

**Authors:** Hao-Li Liu^1^, Han-Yi Hsieh^1^, Li-An Lu^1^, Chiao-Wen Kang^2^, and Chun-Yen Lin^2*^

**Supplementary Figure Captions**

Fig. S1. Heat-shock protein 60 (hsp60) overexpression observed in IHC stain (10X) is induced by microbubble-presented focused ultrasound exposure .

**Supplementary Tables**

## Table S1. Summary of animal experiments. In the experimental groups with 0.6-MPa or 1.4-MPa FUS exposure in the absence of MBs, animals were only evaluated on day 18 after FUS exposure. TIL = tumor infiltrating lymphocyte.

| Group | Control | 0.6-MPa | 0.6-MPa  +MBs | 1.4-Mpa | 1.4-MPa  +MBs |
| --- | --- | --- | --- | --- | --- |
| Tumor Growth | 27 | 18 | 18 | 18 | 18 |
| FITC-dextrans | 4^#^ | 2 | 2 | 2 | 2 |
| TIL infiltration (day 1) | 15 | - | 15 | - | 15 |
| TIL infiltration (day 3) | 10 | - | 10 | - | 10 |
| TIL infiltration (day 18) | 31 | 10 | 11 | 7 | 12 |
| HE | 3 | 3 | 3 | 3 | 4 |
| TUNEL/ hsp60 | 3 | - | - | - | 4 |
| Total | 280 |  |  |  |  |

# Two animals were not injected with FITC-dextrans to serve as sham controls for fluorescent microscopy.

Table S2. Relative FITC-labeled dextran leakage into the tumor regions after FUS exposure. Total area = FITC-fluorescence leakage area in the section; Area fraction = fraction of the FITC-fluorescence area of the whole section. Naive = tumor observation without FUS or FITC-dextran injection. Control = tumor observation with FITC-dextran injection only.

| Group | Relative intensity | Total Area (μm^2^) | Area Fraction (%) |
| --- | --- | --- | --- |
| naive | 41 | 82.4 | 0 |
| control | 36 | 134.8 | 0 |
| 0.6 MPa alone | 121 | 204.4 | 0 |
| 0.6 MPa+MBs | 606 | 2170.2 | 0.4 |
| 1.4 MPa alone | 132 | 379.2 | 0.1 |
| 1.4 MPa+MBs | 1441 | 14383.4 | 2.5 |
